# Assessment and risk reduction of infectious pathogens on chiropractic treatment tables

**DOI:** 10.1186/1746-1340-15-8

**Published:** 2007-06-07

**Authors:** Marion Willard Evans, Jennell Breshears, Alan Campbell, Chris Husbands, Ronald Rupert

**Affiliations:** 1 Associate Professor, Parker College of Chiropractic, Research Institute, 2500 Walnut Hill Lane, Dallas, Texas 75229, USA; 2 Assistant Professor, Parker College of Chiropractic, Research Institute, USA; 3 Professor, Department of Basic Sciences, Microbiology, Parker College of Chiropractic, USA; 4 Laboratory Manager of Pathological and Physiological Sciences, Parker College of Chiropractic, USA; 5 Dean of Research, Parker College of Chiropractic, USA

## Abstract

**Background:**

To investigate the presence of pathogenic microbes on chiropractic treatment tables in one outpatient teaching clinic. Additional aims were to test inexpensive disinfectants on tables that may kill microbes and suggest infection control measures for chiropractic offices, clinics and classrooms. The aim of the study was to assess the presence of pathogenic microbes on treatment tables in one outpatient teaching clinic and determine a simple behavioral model for infection control including table disinfection and accepted hand washing and sanitizing protocols.

**Methods:**

10 treatment tables were selected and sampled for possible microbial flora on face and hand pieces. Samples were cultured on MacConky's agar and mannitol salt agar, labeled and incubated for up to 48 hours. Confirmatory testing of microbes to determine if drug resistant flora were present was performed. Among tables tested, 5 were selected to test disinfectants. One-half of the face piece and 1 hand piece were treated with two different wipes and then post-tested for microbes.

**Results:**

Pathogenic microbes were present on chiropractic treatment tables including methicillin-resistant *Staph aureus*. Simple disinfectants neutralized the pathogens. A rudimentary disinfection procedure and infection control measures are suggested based on the findings.

**Conclusion:**

Pathogenic microbes may be present on chiropractic treatment tables and can be effectively killed with proper disinfecting. Hand washing/sanitizing is an important measure in infection control as is table disinfecting. Rudimentary behavioral changes to improve chiropractic clinic infection control are needed. More comprehensive behavioral models are needed. All teaching clinics and private chiropractic offices should adopt infection control practices including routine table disinfecting and hand sanitizing. Effective measures can be put in place at minimal costs. Accrediting bodies of chiropractic institutions should mandate an infection control plan for member institutions immediately.

## Background

Doctors of chiropractic (DCs) are among the most visited providers of the complementary or alternative practitioners [1]. Most cases seen by DCs involve back pain, neck pain or headache [2]. Approximately one-third of patients with low back pain will consult a DC for this pain [3]. DCs usually employ the use of spinal manipulation to treat the lower back [2]. In order to treat the spine the patient is usually placed prone on a treatment table which has a split facial piece and hand rests. The typical chiropractic table has a cloth or vinyl surface and a changeable face piece paper roll so the patient places his or her face on fresh paper rather than directly on the table.

Bifero and colleagues identified several pathogenic microorganisms on chiropractic tables including methicillin-resistant *Staphylococcus aureus (MRSA) *[4]. This raises concern that chiropractic treatment facilities may not adhere to adequate infection control measures regarding treatment tables. Infection control is more important than ever with the possible threat of pandemic influenza and health care providers are urged to take every precaution to reduce risks in their facilities [5]. The presence of any pathological microbe on chiropractic tables is a potential proxy for pandemic flu and other microbial strains that may spread via droplet or fomite transmission. The aim of this study was to repeat the assessment made by Bifero and colleagues and to identify pathogens on treatment tables in one outpatient teaching clinic if present and to determine if any were a drug-resistant form. Another goal was to test two simple, inexpensive antimicrobials on reducing any threats found on the treatment table surfaces and describe rudimentary infection control measures including table disinfection and proper hand sanitizing.

## Methods

### Sampling

Ten treatment tables were selected for convenience in high use areas of a chiropractic outpatient teaching facility. Inclusion criteria specified tables used in the assessment be covered in a non-porous, vinyl surface and each had a face paper roll to cover the facial surface. Three of the tables had metal hand rests and the remainder had hand pieces covered in vinyl. Cloth fabric tables were specifically excluded from the assessment due to their porous surface and perceived difficulty in obtaining a culture.

A pre-treatment sample was taken from each of the ten tables by applying a sterile swab dipped in sterile saline to the face piece within a 6 cm × 6 cm area with the use of a flexible template to demark a consistent sampling area. The face paper was removed to better access the table surface. The entire contact surface area of each of the hand pieces was swabbed. Individual samples were placed in culture dishes containing MacConky's agar and mannitol salt agar and labeled appropriately.

### Treatment of table surfaces

Immediately after pre-test samples were obtained, five of the tables received a treatment with two sterilizing agents in the following manner; the left half of the face piece received treatment with a pre-packaged alcohol wipe containing 70% isopropyl alcohol and 10% acetone as did the left hand piece, and the right side received treatment with Lysol Brand^® ^sanitizing wipes. Once treated, each was allowed to dry completely and then a post-treatment sample was taken and culture dishes appropriately labeled. All culture dishes were incubated at 35 degrees centigrade for up to 48 hours.

### Isolation and confirmatory testing

Strains were identified as suspected *S. aureus *in the initial incubation and were confirmed with coagulase testing. A second confirmatory test was performed utilizing BBL™ CHROMagar™ (BD Diagnostic Systems) specific for *S. aureus *in which plates turn a mauve color when positive as they hydrolyze the chromogenic substrate in the plate. In order to confirm presence of a *MRSA *strain, a second isolation plate was used (BBL™ CHROMagar™ MRSA) which is *MRSA *specific. Strains of *S. aureus *suspected to be *MRSA *are placed in this medium which contains a cephalosporin. Any mauve color change demonstrates growth despite the presence of the antibiotic which is indicative of *MRSA*. Both CHROMagar ™ plates provide reliable confirmation in 24 hours [6].

## Results

Identification of gram positive (g+) and gram negative (g-) organisms and their differential analysis demonstrated several microbes after 24–48 hours incubation. Two treatment tables contained (g-) organisms and all tables contained at least some (g+) organisms including *S. epidermidis, S. saprophyticus *and *S. aureus*. However, (g-) sub-typing did not successfully confirm a specific organism so this was considered a false-positive detection.

Confirmatory testing for *S. aureus *was positive with both coagulase testing and BBL™ CHROMagar™. Additional testing of the isolates from those positive *S. aureus *plates were subjected to testing with BBL™ CHROMagar™ MRSA and were positive. Post-sanitizing testing demonstrated no pathogenic microbes present on tested tables after use of either of the disinfecting agents.

## Discussion

### Limitations of the study

This study did not quantify bacterial counts as it was felt the presence of any pathogenic microbes in the pre-testing of the tables indicated the need for disinfection protocols. The absence of any standardized protocol in the literature describing reduction of microbial contamination on chiropractic treatment tables was of utmost concern and our methods represent a proposed methodology.

Chiropractic adjusting tables differ from traditional examination tables and represent a special consideration as patients lie prone with their face on a face-piece and with hands in contact with table hand rests. This may require disinfection methods that differ considerably from other medical tables.

An additional limitation was that tables with vinyl face pieces were tested and only 10 were selected to represent all tables in a facility with dozens. This may not be representative of a typical private chiropractic office due to the high patient traffic encountered in teaching clinics. Cloth covered tables were present but excluded as this testing method was most practical for non-porous surfaces.

### Assumptions

This study found *MRSA *on one treatment table. It is assumed that others could contain *MRSA *since only 10 were tested. In addition, an assumption was made that cloth tables could not adequately be sanitized so they were excluded. It is further assumed this issue may be a problem in other chiropractic teaching clinics and even private offices.

### Considerations for controlling for infections in chiropractic offices

Although the face paper on tables does not prevent microbial growth, face paper should be utilized on every treatment table and should be changed after every patient. Paper serves as a barrier to skin secretions, make-up and other discharges from the nose and mouth which left on the table can likely support microbial growth. Direct table surface disinfection should be performed several times per day and when there is a clear clinical indication between routine applications of a disinfectant.

The Centers for Disease Control and Prevention (CDC) suggests surfaces coming in contact with patients be decontaminated [7]. The first step is to clean the surface of any visible soils, organic matter or secretions which could interfere with decontamination procedures. This can be done with soap and water or with any detergent that is safe for surface and patient.

The second consideration for decontamination is the use of a surface disinfectant. The CDC uses the Spaulding Classification system for medical and surgical instruments which has three categories based on the likelihood that the instrument would transfer infection if contaminated; critical surfaces (based on direct patient contact), semi-critical and non-critical [8]. The CDC adds an "environmental" surface to the list to address items not coming into contact with patients such as equipment knobs, carts and handles. Treatment tables should be considered critical or semi-critical surfaces. Several substances are available for commercial use and may include; isopropyl alcohol in concentrations of at least 60–90%, phenolics, sodium hypochlorite including 1:10 dilutions of chlorine bleach, chlorhexidine-containing compounds and ammonium chlorides. In this study, isopropyl alcohol wipes with 70% alcohol (PDI^® ^Nice-pak) were used as were Lysol Brand^® ^Wipes which contained an ammonium chloride solution.

It is proposed that treatment table surfaces be sanitized at the start of the day, mid-day, at the close of the day and any time clinical judgment warrants additional disinfecting. An example would be when a febrile or otherwise notably sick patient is treated on the table or when visible secretions, make-up, or other substances are visible on the surface.

### Hand washing and hand sanitizing

According to the CDC, hand washing and sanitizing may be the single most important factor in reducing spread of infection by an individual or health provider [9]. Any model to reduce exposure of patients and staff must include the issue of hand sanitization, particularly when going from room to room and having direct patient body contact as in the case of most forms of chiropractic manual therapy. However, studies summarizing hand washing activities of health care workers indicate a generally low level of compliance with < 50% in intensive care units [10]. Since chiropractors typically contact patients directly in a hands-on manner, this is concerning.

Guidelines regarding hand washing have been around since 1961 and the CDC suggests that either anti-microbial soaps or waterless sanitizer be used after patient contact to prevent spread of *S. aureus-MRSA*. Plain soaps may remove soils and transient non-pathogenic bacteria, but the CDC states that they may not kill all pathogenic organisms.

Alcohol-based hand sanitizing gel is an acceptable means of hand sanitizing when products contain 70–90% isopropyl alcohol [9]. Some, but not all, contain small amounts of other antimicrobials. Alcohols tend to be effective against several drug-resistant pathogens including *MRSA *and some viruses including hepatitis B and C [9]. The CDC considers alcohol-based sanitizers more effective than standard hand washing and the compliance rates are higher among health personnel, producing less skin drying and dermatitis, when they contain lotions or vitamin E [9]. Hand washing should include soap and should last for at least 20 seconds followed by rinsing in warm water and drying with single-use towels. Sanitizing gels should completely cover hands and fingers on each hand and be allowed to dry and should not be wiped off with a towel [9].

### Placement of hand sanitizer dispensers

Not all clinics or teaching facilities have sinks in each treatment room. Placement of hand sanitizing dispensers is necessary to enhance compliance with infection control protocols. These can be purchased for wall mounting and are relatively inexpensive. They should be placed in each treatment room to make them visible and accessible to all clinicians. The need to facilitate compliance and remove barriers to use cannot be overstated. They may need to be installed strategically in teaching facilities beyond treatment areas since clinicians and students may travel back and forth between those areas. All laboratories where palpation skills are taught or students contact one another should contain sinks or gel dispensers as well. Staff clinicians should be instructed that their behavior is important to vicarious learning opportunities since studies demonstrate hand hygiene is influenced by the visible behaviors of other co-workers [11]. This may be more important when the clinician is the professor in student labs or clinics.

### Other considerations

According to the CDC there are two subtypes of *MRSA *important to health care clinicians [12]. The first is *Hospital Acquired*-*MRSA *(*HA-MRSA*), the second *Community-Acquired MRSA (CA-MRSA)*. Cases of *MRSA *having recent history of hospitalization are suspected to be nosocomial infection. However, those patients who have not recently been hospitalized may have the *CA-MRSA *subtype. Since chiropractic clinicians are not typically seeing patients in a hospital setting, cases may arise from *CA-MRSA*, which has a higher treatment success rate with antibiotics. However, the CDC further suggests the lines are blurring between the subtypes with differentiation becoming more difficult to determine.

Skin lesions such as furuncles, blisters, abscesses and suspected spider-bite lesions should be suspects for *MRSA *infections, particularly in areas where spider-bites are not endemic [13]. The CDC lists several infectious diseases that can be associated with *MRSA *including necrotizing pneumonia and empyema, sepsis syndrome, musculoskeletal infections including pyomyositis and osteomyelitis, necrotizing fasciitis, purpura fulminans and disseminated infections with septic emboli [12].

This investigation focused on those pathogens found on non-porous treatment table surfaces. Porous surfaces could represent an even greater concern including cloth surfaces such as fabric covered treatment tables or chairs [14]. Patient gowns, towels, hot pack covers, traction harnesses and other devices coming in contact with a patient's skin could be sources of microbial pathogens and should be washed with hot water and detergent before the next patient use. Items soiled with body fluids, especially by patients who are known to be febrile or sick (including tissues or table face-paper) should be handled only with gloves and disposed of in an appropriate manner or treated with an appropriate germicide. Biohazards should be placed in biohazard containers and disposed of under established guidelines. Airborne pathogens may also need to be considered in some areas of the country where diseases like tuberculosis are prevalent.

### Infection control as risk management

The Chiropractic and Osteopathic College of Australasia has a document on risk management that includes a comprehensive section on infection control [15]. Important points to consider from this manual include suggestions that hand washing should occur before and after any patient contacts, that gloves should be worn when there is a history of skin lesions or visible lesions present on the patient and that tables need to be covered in non-porus material such as vinyl. This guide suggests tables be wiped with a disinfectant after each patient contact.

Certainly all considerations given to handling blood products, human body fluids and other biohazards should go without saying. However, this manual is very detailed on infection control from a risk management perspective and this should not be understated. Knowingly or unknowingly spreading infection to patients, staff and family when risk reduction measures are known is unacceptable and health care providers need to take liability risk into consideration as well.

## Conclusion

A systematic infection control protocol may not be in place for the chiropractic profession and is clearly needed. The suggestions here are rudimentary but a necessary start. To our knowledge, no studies indicate the potential risk of acquiring an infection from a chiropractic treatment table surface. Future research should determine the relative risk associated with treatment on tables where inadequate infection control measures are an issue. Studies that quantify the amount of pathogenic microbes on treatment surfaces should be considered as risk may hinge on quantity.

Additional testing of tables used in chiropractic offices that are made of porous, cloth materials may be needed. These may not be susceptible to disinfecting measures described in this study and could harbor other pathogens such as mold spores, dust mites and even pathogenic bacteria and viruses.

Accrediting bodies of all colleges and schools that utilize manual therapy should immediately require that infection control measures be practiced for adequate patient and clinician safety similar to what has been developed in Australia. Active and passive methods of infection control should be investigated to enhance safety in chiropractic practices and teaching institutions, including routine surveillance programs.

## Abbreviations

CA-MRSA-Community-acquired methicillin-resistant Staphlococcus aureus

CDC-US Centers for Disease Control and Prevention

DC-Doctor of Chiropractic

G+-Gram positive staining

G- -Gram negative staining

HA-MRSA-Hospital-acquired methicillin-resistant Staphlococcus aureus

MRSA-Methicillin-resistant Staphlococcus aureus

## Competing interests

The author(s) declare that they have no competing interests.

## Authors' contributions

MWE, AC and RR contributed to the study design of the project. MWE, JB, AC collected samples and AC and CH analyzed microbiological samples and sub-samples. All authors contributed to and approved the final manuscript.

## Funding sources and conflicts of interest

The funding for this project was internal through Parker Research Institute and the authors declare no conflicts of interest.
